# Circulating miR-19a and miR-205 in Serum May Predict the Sensitivity of Luminal A Subtype of Breast Cancer Patients to Neoadjuvant Chemotherapy with Epirubicin Plus Paclitaxel

**DOI:** 10.1371/journal.pone.0104870

**Published:** 2014-08-19

**Authors:** Qian Li, Mei Liu, Fei Ma, Yang Luo, Ruigang Cai, Liming Wang, Ningzhi Xu, Binghe Xu

**Affiliations:** 1 Department of Medical Oncology, Cancer Institute and Cancer Hospital, Chinese Academy of Medical Sciences and Peking Union Medical College, Beijing, PR China; 2 Laboratory of Cell and Molecular Biology & State Key Laboratory of Molecular Oncology, Cancer Institute and Cancer Hospital, Chinese Academy of Medical Sciences and Peking Union Medical College, Beijing, PR China; 3 Department of Abdominal Surgery, Cancer Institute and Cancer Hospital, Chinese Academy of Medical Sciences and Peking Union Medical College, Beijing, PR China; 4 Department of Tumor Chemotherapy and Radiation Sickness in Peking University Third Hospital, Beijing, China; University of Salerno, Faculty of Medicine and Surgery, Italy

## Abstract

**Background:**

The luminal A subtype of breast cancer has a good prognosis and is sensitive to endocrine therapy but is less sensitive to chemotherapy. It is necessary to identify biomarkers to predict chemosensitivity and avoid over-treatment. We hypothesized that miRNAs in the serum might be associated with chemosensitivity.

**Methods:**

Sixty-eight breast cancer patients received neoadjuvant chemotherapy with epirubicin plus paclitaxel. The serum of the patients was collected before chemotherapy and stored at −80°C. The samples were classified into two groups in term of the chemosensitivity. We identified the differential expression patterns of miRNAs between the chemotherapy sensitive and resistant groups using microRNA profiling. Four miRNAs that were differentially expressed between the two groups were further validated in another 56 samples. We created a model fitting formula and a receiver operating characteristics (ROC) curve using logistic regression analysis to evaluate the prediction potency.

**Results:**

We identified 8 miRNAs differentially expressed between the two groups: 6 miRNAs were up-regulated, and 2 miRNAs were down-regulated in the resistant group compared with the sensitive group. The expression of miR-19a and miR-205 were determined to have significant differences between the two groups (P<0.05). A predictive model of these two miRNAs was created by the logistic regression analysis. The probability of this model was 89.71%. Based on the ROC curve, the specificity was 75.00%, and the sensitivity was 81.25%.

**Conclusions:**

The combination of miR-19a and miR-205 in the serum may predict the chemosensitivity of luminal A subtype of breast cancer to epirubicin plus paclitaxel neoadjuvant chemotherapy.

## Introduction

The luminal A subtype of breast cancer is a type of breast cancer that is ER (estrogen receptor) and/or PR (progesterone receptor) positive, Her2-negative, and Ki67<14%. This type of breast cancer has a relatively better prognosis but is less sensitive to chemotherapy compared to other subtypes of breast cancer [1]. Clinically, a patient's risk of recurrence for this type of breast cancer is usually determined based on the number of the histological grade, lymph node metastases, vascular tumor suppository, and the primary tumor size. For patients with a higher recurrence risk, chemotherapy is generally recommended. For patients with a relatively low risk, endocrine therapy is prescribed instead. Certain luminal A subtype patients, even with a high risk, are not sensitive to chemotherapy; therefore, it is necessary to find a predictive marker to select the sensitive breast cancer patients with luminal A subtype to receive chemotherapy and to avoid over-treatment of the resistant group.

MicroRNA (miRNA) is a type of endogenous non-coding RNA (ncRNA). They are responsible for post-transcriptional regulation and participate in nearly all biological processes. Expression profiling has shown that miRNAs can distinguish between normal breast and tumor tissues [2] and can be used to classify poorly differentiated breast tumors into subtypes [3]. In recent years, circulating miRNAs have become promising biomarkers based on their stability in harsh conditions and on their non-invasive testing and feasibility in clinical practices. Current reports showed that serum microRNA expression could be used as an early marker for determining the breast cancer risk [4]. The concentrations of some circulating microRNAs (miR-17, miR-34a, miR-155, and miR-373) in human breast cancer have been correlated with tumor development and progression. Aberrant miRNA expression may be involved in drug resistance to various chemotherapeutic agents in breast cancer [5]. To date, the predictive value of circulating miRNAs for the treatment outcomes in luminal A subtype of breast cancer has not been investigated.

In this study, we hope to investigate the value of miRNAs in predicting the effect in luminal A subtype of breast cancer treatment. MicroRNAs from peripharal blood were detected. The miRNAs whose expression was significantly different between sensitive and resistant groups were selected and validated in larger sample size. Finally, a predictive model of miRNAs was created, which could potentially be used to predict chemosensitivity in luminal A subtype of breast cancer.

## Materials and Methods

### Patients

This study recruited 68 patients with stage IIa-IIIc luminal A subtype breast cancer. The clinical data of the patients who were treated with neoadjuvant chemotherapy from January 2012 to March 2013 were collected. All the patients received neoadjuvant chemotherapy of epirubicin (75 mg/m^2^, q21 d) on day 1 and paclitaxel (175 mg/m^2^, q21 d) on day 2 after undergoing core needle biopsies of their primary tumors and determination of the estrogen receptor (ER), progesterone receptor (PR) and HER2 status by immunohistochemical (IHC) examinations. Treatment was continued in the absence of unacceptable toxicity or disease progression for a maximum of six cycles given every 21 days. All of the patients were followed-up until June 2013.

The residual tumor burden was measured by CT to evaluate the tumor response before surgical resection. The pathologic response was assessed according to the Miller and Payne (MP) histopathology scoring system [6].Grade 1: no reduction; grade 2: minor loss (≤30%); grade 3: some loss (30%–90%); grade 4: marked loss (>90%); grade 5: no residual invasive cancer. According to the chemosensitivity, the patients were stratified into two subgroups, including a sensitive group (>grade 2) and a resistant group (≤grade 2). There was a balanced distribution in the sensitive and resistant groups of the age, menstrual status, tumor size and lymph node metastasis (Table 1).

**Table 1:** Clinical pathological factors between the sensitive and resistant groups.

Factors	resistant (30)	sensitive (38)	*P* value
**stage** *			NS*
II	5	6	
III	25	32	
**Age (median)**	48	49	NS
**Histologic stage**			NS
I	2	2	
II	20	26	
III	8	10	
**T stage** *			NS
T1	2	3	
T2	10	13	
T3	12	13	
T4	6	9	
**menopause**			NS
Yes	14	17	
No	16	21	

*TNM stage is according to the AJCC seventh edition.

*NS: P≥0.05.

### Ethics statement

This study was approved by the medical ethics committee of Cancer Institute and Cancer Hospital, Chinese Academy of Medical Science and Peking Union Medical College, and all the participants signed written informed consent forms.

### Serum collection

All serum samples were collected before the patients had received neoadjuvant chemotherapy. Two milliliters of venous blood were collected from the 68 breast cancer patients. To harvest cell-free serum, the blood was drawn into a sterile tube without anticoagulant. After leaving the tube in standing position for 20 min, samples were centrifuged at a speed of 3000 rpm for 5 min, and the supernatant serum was quickly removed and stored immediately at −80°C until analysis.

### TaqMan Real-time PCR microRNA Array

In the screening phase, we performed TaqMan Real-time PCR microRNA Arrays to identify the differentially expressed miRNAs from the two pooled serum samples (6 from the sensitive group compared with 6 from the resistant group). Serum (600 µl) from each pooled sample was used. The miRNA was isolated using TRIzol LS Reagent (Invitrogen). The concentration and quality of RNA were determined by using NanoDrop ND-2000 (Thermo Scientific, Wilmington, DE, USA). Megaplex RT reactions and pre-amplification reactions were run according to the manufacturer's protocol (Applied Biosystems, Foster City, CA, USA). The quantitative miRNA expression data were acquired using the ABI 7900HT SDS software (Applied Biosystems)(settings: automatic baseline, threshold 0.2). Because U6 is not suitable for microRNA detection in serum, we selected miR-484 as our endogenous control [7]. The fold changes in gene expression were calculated using the 2^−ΔΔC_T_^ method [8]. ΔC_T_ = C_T miR-of-interest_−C_T miR-484_. ΔΔC_T_ = ΔC_T resistant_−ΔC_T sensitive_.

### Validation

In the validation phase, four candidate miRNAs identified by Array A were further characterized. Serum samples from 56 breast cancer patients (32 cases from the sensitive group and 24 cases from the drug-resistant group with well-balanced clinical pathologic features) were analyzed by using SYBR-based real-time PCR (Quantobio Technology, Beijing, China). The QuantoBio Total RNA Isolation Kit was used to isolate the RNA from the serum samples (Quantobio Technology, Beijing, China). Each sample was eluted with 40 µl RNase-free water. The concentration of RNA extracted from the serum samples ranged from 4.9 ng/µl to 44.7 ng/µl. The miR-Quanto System is composed with three reaction steps to convert and quantify levels of miRNA expression. Firstly, a polyadenine tail is attached to miRNA at their 3′ end. This is followed by a retro-transcription step that converts miRNA into cDNA and attaches a universal DNA tag at the 5′ end of synthesized cDNA. After the first strands of cDNA synthesized, qPCR was performed by using the miRNA specific forward primer and a reverse universal primer mix. The data were normalized using the external controls Quanto EC1 and Quanto EC2. Both Quanto EC1 and Quanto EC2 were synthetic miRNAs with 24 bp and 21 bp, respectively. Quanto EC1 that monitors the extraction process was added when the serum samples were extracted and Quanto EC2 that monitors the cDNA synthesis and qPCR reaction was added in the RNA samples. All of the reactions were performed according to the manufacturer's instructions.

### Statistical analysis

SPSS16.0 software was used for the statistical analysis. Statistical descriptions were used to describe the clinical pathological features, and the t test (Student's t test) was used to analyze the measurement data. The Mann-Whitney U in nonparametric test was used to analyze the continuous variables. The P value was bilaterally tested, and values less than 0.05 were regarded as statistically significant.

## Results

### Screening phase

To obtain sufficient amount of cDNA for TaqMan Real-time PCR microRNA Array, pre-amplification step using TaqMan PreAmp MasterMix was added. Because U6 is not suitable for microRNA detection in serum [9], we selected miR-484 as our endogenous control [7]. The miRNAs satisfying the following criteria were selected for further confirmation by individual qRT-PCR: (1) having a 15–30 C_T_ value by Array in the two pools, (2) showing a 5-fold altered expression by Array. After being analyzed, 8 miRNAs satisfied the criteria. Of those 8 miRNAs, 6 were up-regulated and 2 were down-regulated in the resistant group compared with the sensitive group (Table 2).

**Table 2:** Eight miRNAs with significant differences between the resistant and sensitive groups.

	miRNA	Fold change
		resistant/sensitive
Array A	hsa-miR-19a	6.60
	hsa-miR-28	8.73
	hsa-miR-205	7.65
	hsa-miR-375	8.71
	hsa-miR-194	−39.00
	hsa-let-7b	−12.32
Array B	hsa-miR-1290	5.16
	hsa-miR-1274A	5.73

### Validation

**Figure 1:**
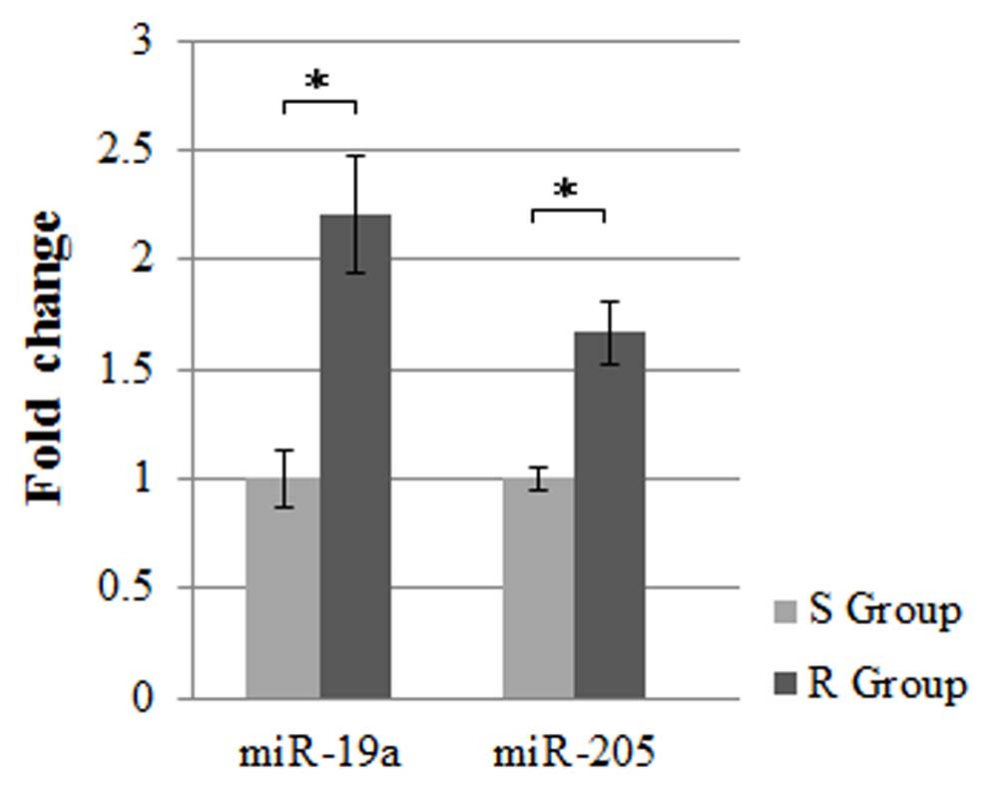
Differences in the microRNAs expression levels between the sensitive and resistant groups. The expression level of serum miR-19a and miR-205 in luminal A breast cancer was examined by qRT-PCR. The relative levels of miR-19a and miR-205 normalized to its expression in sensitive group, respectively (set as 1). Mean± s.d. R: resistant, S: sensitive. *p<0.01.

**Figure 2:**
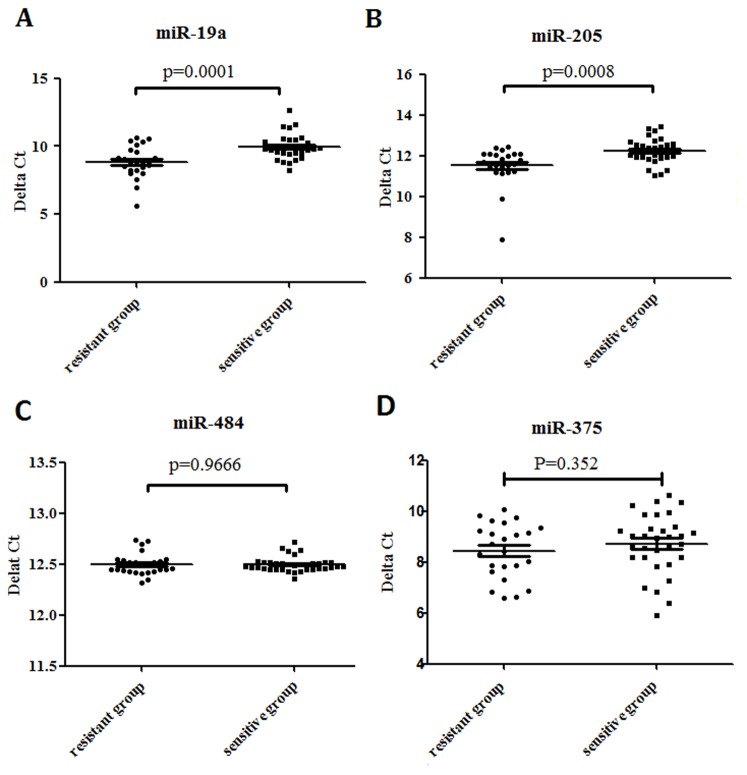
The scatter diagram of circulating miR-19a, miR-205, miR-484 and miR-375 in 56 luminal A breast cancer patients. Quantitative real-time PCR analysis of miR-19a, miR-205, miR-484 and miR-375 in resistant group (n = 24) and sensitive group (n = 32). Mean± s.d.

The stem-loop RT-PCR based TaqMan MicroRNA Arrays (Applied Biosystems) representing 762 mature miRNAs in a two-card set of arrays (Array A and Array B) was used. Array A focuses on the more highly characterized miRNAs, and Array B contains many of the more recently discovered miRNAs and the miR* sequences. Here, although we used the same amount of total RNA to do the RT and pre-amplification reaction in Array A and Array B, the level of miR-484 could only be detected in Array A card which indicated that Array A might be better in accuracy. That's why we preferred Array A to Array B. After excluding 2 miRNAs of Array B, we selected 4 miRNAs to further validate in the other 56 patients based on the mining of public literatures that have been reported by different study cohorts of breast cancer [10]–[16]. We also based on the PubMed hits when particular miRNA was combined with the keyword “breast cancer”. The 4 miRNAs were let-7b, miR-19a, miR-205 and miR-375. In order to validate the reliability and accuracy of miR-484 as an endogenous control in the screening phase, we also detected its level in the validation phase. We used a poly(A) miRNA-based real-time PCR to detect these 5 miRNAs. As shown in Table 3 and Figure 1, the expression of miR-19a and miR-205 increased more in the resistant group compared with the sensitive group (p<0.05). The scatter diagram of miR-19a, miR-205, miR-484 and miR-375 in all patients was shown in Figure 2. The result showed that miR-484, as an endogenous control, had no differences between the two groups (p>0.05, Figure 2C). The variation tendencies of let-7b were opposite to the result of the miRNA Array. The level of miR-375 has no significant differences between the two groups (p>0.05).

**Table 3:** Variation differences of 4 miRNAs.

miRNA	*2^−ΔΔC_T_^ resistant/sensitive	P value
miR-19a	2.21	0.004
miR-205	1.66	0.000
let-7b	1.52	0.001
miR-375	1.22	0.352

* 2^−ΔΔC_T_^ refers to the ΔC_T_ = C_T miR-of-interest_−C_T QuantoEC1/QuantoEC2_. ΔΔC_T_ = ΔC_T resistant_−ΔC_T sensitive_.

### Modeling

**Figure 3:**
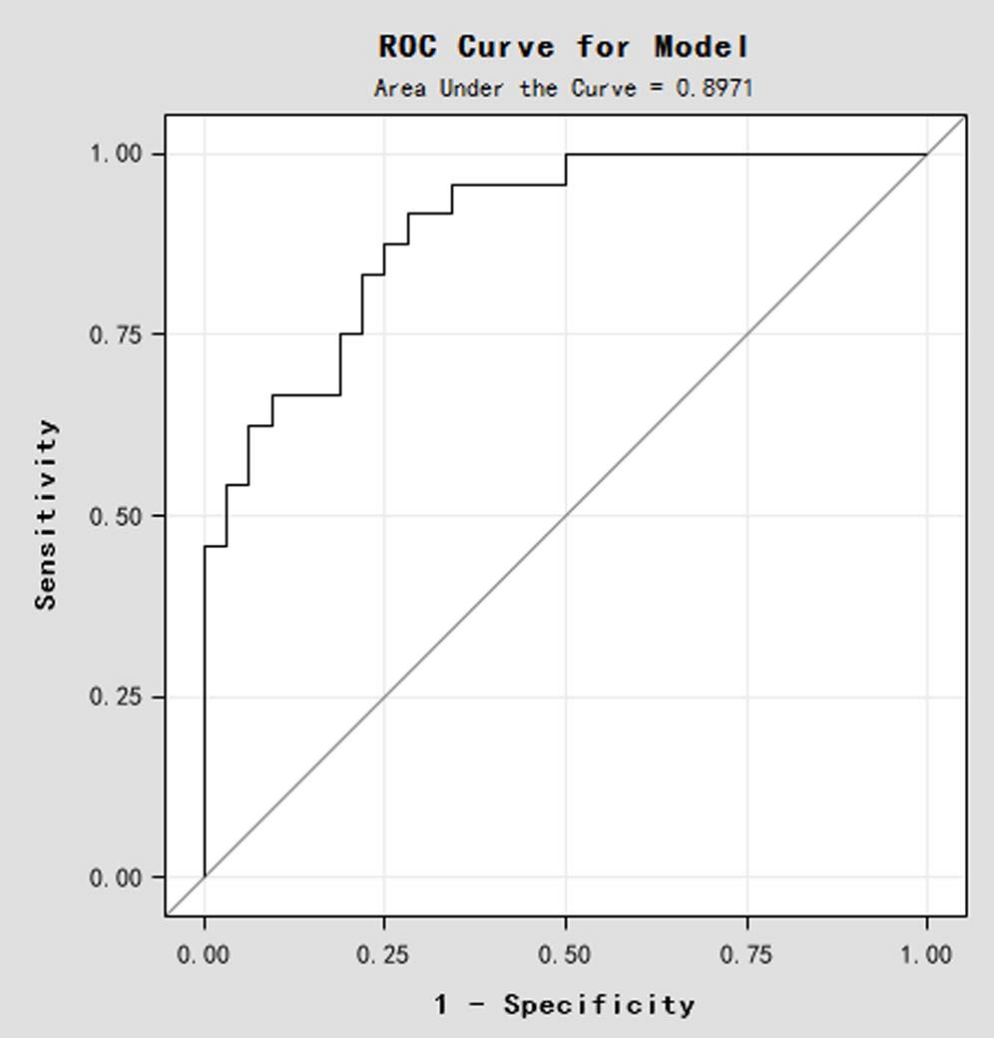
The predictive value of miR-19a and miR-205 expression regarding luminal A breast cancer patients' treatment responses. A ROC curve analysis for miR-19a and miR-205 expression was performed to assess the luminal A breast cancer treatment response.

Firstly, univariate and multivariate Cox proportional hazards regression analysis were used to identify the association of chemotherapy response and the 3 miRNAs. As shown in table 4, multivariate analysis revealed that miR-19a and miR-205 were the independent predict factors associated with chemotherapy response. Therefore, a predictive model of miR-19a and miR-205 was created by the logistic regression analysis as: p = EXP(47.7312−1.5637×ΔC_TmiR-19a_−2.7708×ΔC_TmiR-205a_)/[1+EXP(47.7312−1.5637×ΔC_TmiR-19a_−2.7708×ΔC_TmiR-205a_)]. The probability of this model was 89.71%. To evaluate the diagnostic value, we used the ROC (receiver operating characteristics) curve to analyze the sensitivity and specificity of this model (Figure 3). The ROC curve with the 2 miRNAs was more potent than the other combinations. The specificity and sensitivity were 75.00% and 81.25%, respectively.

**Table 4:** Univariate and Multivariate Cox proportional hazards regression analysis of miRNAs in relation to chemotherapy response.

Variable	Chemotherapy Response			
	Univariate analysis		Multivariate analysis	
	HR(95%CI)	p value	HR(95%CI)	p value
miR-19a	3.565(1.631–7.794)	0.001	4.777(1.743–13.088)	0.002
miR-205	6.575(1.893–22.842)	0.003	15.974(2.539–100.511)	0.003
let-7b	4.316(1.435–12.980)	0.009		

HR, hazard ratio; CI, confidence interval; miR, microRNA.

## Discussion

Neoadjuvant chemotherapy was recommended for inoperable locally advanced tumors, in order to make the unresectable tumors can be removed. However, it has been controversial as to whether neoadjuvant chemotherapy should be given in luminal A subtype. Some studies suggested that neoadjuvant treatment should be given based on tumor subtype instead of tumor size [17]. Meanwhile, another group of opinion was that ER positive was not the criteria to decide whether chemotherapy should be given or not. Instead, chemotherapy should be given to the patients with positive axillary lymph nodes, regardless of their tumor biology [18]. According to the NCCN (National Comprehensive Cancer Network) guidelines, chemotherapy is required for high risk luminal A subtype patients. Based on the relatively low sensitivity to chemotherapy of the luminal A subtype, some patients are resistant to chemotherapy even if they are high risk of recurrence. Screening out the patients who will be resistant to chemotherapy is a reasonable way to avoid ineffective chemotherapy.

In recent years, the clinical value of miRNAs has been assessed because of its tumor-specific expression and stability in tissues and in the circulation. Circulating miRNAs can be predictive as well as prognostic biomarkers in various cancers [19], including breast cancer [20]–[22]. Further study indicated that miRNAs expression pattern is diverse in different breast cancer subtypes, but no study to date has been reported for the application of miRNAs to patients with luminal A breast cancer that received neoadjuvant treatment. Based on the efficacy of neoadjuvant chemotherapy, we classified all the patients into sensitive group and resistant group. In some subtypes of breast cancer, it should be grouped by pathologic complete response (pCR). In this study, pCR and Partial Response (PR) patients were included as the sensitive group because of two reasons. First, it was reported that the pCR rate was only 1.5–10% in the luminal A subtype [23], so it was difficult to balance the pCR group with the non-pCR group because of the wide gap of numbers between the resistant and sensitive groups. Second, pCR has prognostic value only in more aggressive breast cancer subtype such as Luminal B (HER2-negative), HER2-positive (non-luminal) and triple-negative breast cancers, but not in luminal A subtype [24]. Therefore, the pCR rate in the luminal A subtype is less meaningful than in other subtypes. Here, we tried to use a pathological evaluation system (MP) to make a relatively more credible evaluation result. This attempt may need further validation for the long-term survival.

Endogenous control(s) are essential to normalizing the differences between the groups. An ideal endogenous control should have stable expression, similar storage stability, and similar extraction and quantification results [25]. A consensus regarding suitable endogenous control in the detection of circulating miRNAs has not been found, and it is also not recommended to use the same endogenous control in tissues and in blood, so researchers tend to select the endogenous control from the published literature or validated controls. In our study, we selected miR-484 as the endogenous control to analyze the array data because of the previous report [7]. In order to validate the reliability and accuracy of miR-484 as an endogenous control in the screening phase, we detected its level in 56 serum samples and analyzed the relative expression level by using the external controls Quanto EC1 and Quanto EC2 in the validation phase. There was no statistically significant difference of miR-484 between the resistant group and sensitive group. Although we detected the expression levels of miRNAs in the screening and validation phases by using two different methods, the consistency of miR-484 between the sensitive and resistant group may provide clue to support the reliability of our validation results.

In this study, we identified two miRNAs in the serum for predicting the chemosensitivity in luminal A subtype breast cancer patients. To our knowledge, this is the first study using miRNAs in the serum to predict the chemosensitivity of luminal A subtype of breast cancer to epirubicin plus paclitaxel neoadjuvant chemotherapy. Previous reports showed that PTEN was a direct target of miR-19a and loss of PTEN could activate PI3K/Akt pathway which can elevate MDR-1 [26], MRP1 [27] and BCRP [28]. Then, the increase of mutidrug resistance proteins may be one of the causes to lead resistance to chemotherapy. Besides, androgen receptor could up-regulate miR-19a and androgen receptor positive breast cancer has relatively low chemosensitivity [29]. A recent study showed that breast cancer tumor tissues indeed produced miR-19a and that the serum levels of miR-19a of high-risk patients drop less rapidly than low-risk patients [15]. miR-205 is found exclusively in normal ducts and lobular myoepithelial cells of the breast but is significantly reduced in breast tumor tissues [30], [31]. In present study, we reported that increased concentrations of miR-205 were found in serum of the resistant patients compared with the sensitive patients. Thus, serum miR-205 maybe served as a marker to present disease severity-dependent change of breast cancer patients. Furthermore, miR-205 had been reported overexpressed in androgen receptor positive TNBC tumor cells but with low expression in androgen negative TNBC [32]. However, the molecular mechanisms of miR-19a and miR-205 on chemotherapy sensitivity of breast cancer need to be further elucidated.

The study has some limitations. We have not obtained the long-term survival data of all the patients. It remains unknown as to whether the patients who were sensitive to chemotherapy in our study could live longer. In addition, the sample size is not large enough to validate the final model. Although long-term follow-up data and a larger sample size are needed for further validation, our data shed light on understanding the value of circulating miRNAs in predicting the chemosensitivity of the luminal A subtype.

Taken together, the present study revealed that miR-19a and miR-205 were highly expressed in breast cancer patients with the luminal A subtype who are resistant to the epirubicin plus paclitaxel regimen. The modeling of the two combined miRNAs, miR-19a and miR-205, may be useful in predicting the chemosensitivity of the luminal A subtype.
