# Correction: Circulating miR-19a and miR-205 in Serum May Predict the Sensitivity of Luminal A Subtype of Breast Cancer Patients to Neoadjuvant Chemotherapy with Epirubicin Plus Paclitaxel

**DOI:** 10.1371/journal.pone.0136826

**Published:** 2015-08-25

**Authors:** Qian Li, Mei Liu, Fei Ma, Yang Luo, Ruigang Cai, Liming Wang, Ningzhi Xu, Binghe Xu

There is an error in reference 29. The correct reference is: Mo W, Zhang J, Li X, Meng D, Gao Y, Yang S, et al. (2013) Identification of Novel AR-Targeted MicroRNAs Mediating Androgen Signalling through Critical Pathways to Regulate Cell Viability in Prostate Cancer. PLoS ONE 8(2): e56592.
